# RETRACTION: Effects of Platelet‐Rich Fibrin on Post‐Extraction Wound Healing and Wound Pain: A Meta‐Analysis

**DOI:** 10.1111/iwj.70442

**Published:** 2025-03-26

## Abstract

**RETRACTION:** YuanY. , XuB. , YangJ. , and WuM. , “Effects of Platelet‐Rich Fibrin on Post‐Extraction Wound Healing and Wound Pain: A Meta‐Analysis,” International Wound Journal 21, no. 2 (2024): e14654, 10.1111/iwj.14654.PMC1083091842053019

The above article, published online on 31 January 2024, in Wiley Online Library (http://onlinelibrary.wiley.com/), has been retracted by agreement between the journal Editor in Chief, Professor Keith Harding; and John Wiley & Sons Ltd. Following an investigation by the publisher, all parties have concluded that this article was accepted solely on the basis of a compromised peer review process. The editors have therefore decided to retract the article. The authors did not respond to our notice regarding the retraction.

**RETRACTION:** Y. Yuan , B. Xu , J. Yang , and M. Wu , “Effects of Platelet‐Rich Fibrin on Post‐Extraction Wound Healing and Wound Pain: A Meta‐Analysis,” International Wound Journal 21, no. 2 (2024): e14654, 10.1111/iwj.14654.PMC1083091842053019

The above article, published online on 31 January 2024, in Wiley Online Library (http://onlinelibrary.wiley.com/), has been retracted by agreement between the journal Editor in Chief, Professor Keith Harding; and John Wiley & Sons Ltd. Following an investigation by the publisher, all parties have concluded that this article was accepted solely on the basis of a compromised peer review process. The editors have therefore decided to retract the article. The authors did not respond to our notice regarding the retraction.

